# Patient Preferences for Lung Cancer Treatment: A Qualitative Study Protocol Among Advanced Lung Cancer Patients

**DOI:** 10.3389/fpubh.2021.622154

**Published:** 2021-02-05

**Authors:** Ilaria Durosini, Rosanne Janssens, Reinhard Arnou, Jorien Veldwijk, Meredith Y. Smith, Dario Monzani, Ian Smith, Giulia Galli, Marina Garassino, Eva G. Katz, Luca Bailo, Evelyne Louis, Marie Vandevelde, Kristiaan Nackaerts, G. Ardine de Wit, Gabriella Pravettoni, Isabelle Huys

**Affiliations:** ^1^Applied Research Division for Cognitive and Psychological Science, IEO, European Institute of Oncology IRCCS, Milan, Italy; ^2^Department of Pharmaceutical and Pharmacological Sciences, KU Leuven, Leuven, Belgium; ^3^School of Health Policy & Management, Erasmus University Rotterdam, Rotterdam, Netherlands; ^4^Julius Center for Health Sciences and Primary Care, University Medical Center Utrecht, Utrecht University, Utrecht, Netherlands; ^5^Alexion Pharmaceuticals, Inc., University of Southern California School of Pharmacy, Los Angeles, CA, United States; ^6^Department of Oncology and Hemato-Oncology, University of Milan, Milan, Italy; ^7^Unit of Thoracic Oncology, Medical Oncology Department, Fondazione IRCCS Istituto Nazionale dei Tumori, Milan, Italy; ^8^Janssen Research and Development, Raritan, NJ, United States; ^9^Department of Pneumology/Respiratory Oncology, University Hospital Leuven, KU Leuven, Leuven, Belgium

**Keywords:** patient preferences, drug decision-making, lung cancer, drug development, patient-centered research, patient involvement, focus group discussion, nominal group technique

## Abstract

**Introduction:** Lung cancer is the deadliest and most prevalent cancer worldwide. Lung cancer treatments have different characteristics and are associated with a range of benefits and side effects for patients. Such differences may raise uncertainty among drug developers, regulators, payers, and clinicians regarding the value of these treatment effects to patients. The value of conducting patient preference studies (using qualitative and/or quantitative methods) for benefits and side effects of different treatment options has been recognized by healthcare stakeholders, such as drug developers, regulators, health technology assessment bodies, and clinicians. However, evidence-based guidelines on how and when to conduct and use these studies in drug decision-making are lacking. As part of the Innovative Medicines Initiative PREFER project, we developed a protocol for a qualitative study that aims to understand which treatment characteristics are most important to lung cancer patients and to develop attributes and levels for inclusion in a subsequent quantitative preference survey.

**Methods:** The study protocol specifies a four-phased approach: (i) a scoping literature review of published literature, (ii) four focus group discussions with stage III and IV Non-Small Cell Lung Cancer patients, (iii) two nominal group discussions with stage III and IV Non-Small Cell Lung Cancer patients, and (iv) multi-stakeholder discussions involving clinicians and preference experts.

**Discussion:** This protocol outlines methodological and practical steps as to how qualitative research can be applied to identify and develop attributes and levels for inclusion in patient preference studies aiming to inform decisions across the drug life cycle. The results of this study are intended to inform a subsequent quantitative preference survey that assesses patient trade-offs regarding lung cancer treatment options. This protocol may assist researchers, drug developers, and decision-makers in designing qualitative studies to understand which treatment aspects are most valued by patients in drug development, regulation, and reimbursement.

## 1. Introduction

Lung cancer is the deadliest and most prevalent cancer worldwide (1–3). The *World Health Organization* (WHO) estimates that lung cancer death rates will continue to rise, mainly as a result of some lifestyle and environmental factors such as cigarette smoking (4, 5). Lung cancer incidence and mortality rates are highest in developed countries and peak between 65 and 84 years (6). There are two main forms of lung cancer: *Non-Small Cell Lung Cancer* (NSCLC) and *Small Cell Lung Cancer* (SCLC). NSCLC is the most common type of lung cancer, accounting for 85% of patients. Frequent symptoms include cough, dyspnea, hemoptysis, and chest pain (7). Clinical outcomes for NSCLC depend on the stage at the time of diagnosis. Often, patients are diagnosed with NSCLC in an advanced-stage, resulting in a poor prognosis and a 5-year survival rate below 20% (7–10). Treatment options for lung cancer vary widely according to disease stage and characteristics. (Locally) advanced NSCLC patients may have received several treatments, in combination or sequence, including chemotherapy, targeted therapy, immunotherapy, surgery, and radiation therapy (6, 11–18).

Lung cancer treatments are associated with different treatment attributes (or features), such as benefits (e.g., in terms of progression-free survival, overall survival, response rate), risks (side effects such as fatigue and hair loss), route of administration and treatment schedule. Such differences may raise uncertainty among drug developers, regulators, payers, and clinicians regarding the value of these treatment attributes to patients. Patient preference studies provide evidence from patients on what treatment attributes are important, how important these attributes are, and which trade-offs patients are willing to make between attributes (19).

Recent research highlights that results from studies that investigate patients' preferences, called *patient preference studies*, could inform decisions across the drug life cycle. Using patient preference studies to inform these decisions may improve the decision-making process and patients' experience with the treatment, leading to better outcomes and better use of resources (20–22). The drug life cycle is the process of developing a drug and bringing it to patients. It consists of the following subsequent stages and decisions, all of which may be informed by patient preference studies: discovery, preclinical development, clinical development, marketing authorization, *Health Technology Assessment* (HTA), pricing, reimbursement and post marketing. Stakeholders involved in the drug life cycle—HTA bodies, payers, academics, patients and patient organizations, physicians, industry, and regulators—are exploring how to design, conduct and use patient preference studies to inform drug decision-making (19, 23–25).

An important step in the design of patient preference studies is the selection of the attributes and attribute levels further investigated in the quantitative phase of the preference study. Attributes may include different types of benefits and risks associated with treatments and other clinical and non-clinical aspects that can influence desirability or acceptability of treatments to patients (26). Authors have also described attributes as characteristics or features. Examples of attributes are mode of treatment administration, treatment benefits (e.g., survival or tumor reduction in the case of cancer) or treatment risks (side effects such as nausea, diarrhea). Attribute levels are the values or categories used to characterize the performance of a treatment under each attribute in a preference survey. As qualitative methods, such as focus groups, allow to examine patients' experiences and enable sensitive topics to be discussed, their use for identifying the treatment attributes and levels is being increasingly recognized. Attributes and levels developed through qualitative methods have been described to be richer, and qualitative methods with patients reduce the potential for misspecification of attributes through overreliance on the views of experts and researchers (27, 28).

However, detailed information on methodological and practical questions as to how to use qualitative research to identify and develop the attributes and levels for inclusion in patient preference studies aiming to inform decisions across the drug life cycle is currently lacking. This absence of methodological consensus and practical guidance underscores the importance of testing qualitative methods and reporting on them in the published literature.

This paper describes the protocol of a qualitative study that aims to understand which treatment characteristics are most important to advanced lung cancer patients and to identify attributes and levels for inclusion in a subsequent quantitative preference survey. This study will illustrate the value of using a qualitative approach with patients to identify preferred treatment characteristics and develop attributes from these characteristics.

The results from applying this study protocol will be used to develop a subsequent preference survey that quantifies: (i) the relative importance of the attributes and attribute levels identified in this qualitative phase among a larger group of patients and (ii) the trade-offs patients are willing to make between lung cancer treatments that vary with respect to these attributes and levels.

This qualitative study is conducted as part of the *Innovative Medicines Initiative* (IMI) *Patient Preferences in Benefit-Risk Assessments during the Drug Life Cycle* (PREFER) project. PREFER will develop evidence-based recommendations to guide industry, regulatory authorities, and HTA bodies (including reimbursement agencies and payers) on how patient preference studies should be performed and used to inform decision-making throughout the drug life cycle (29, 30). Taking attention to patients' preferences in the drug life cycle becomes increasingly important not only for companies that develop new medical products, but also for the authorities that regulate, assess, and decide which products are safe, effective, well-tolerated, and cost-effective (31–33). Exploring patient preferences may provide information on medical products from the patients' perspective (such as information on the importance to patients of clinical outcomes and safety issues) and can lead to patient-centric decision making processes (34). More specifically, patient preference studies could be included in the following decisions in the drug life cycle: (i) industry decisions on which medical product to develop, based on the unmet needs of patients, as revealed through preference studies, (ii) decisions on which clinical trial endpoints to include in clinical trials, and (iii) value assessments concerning the clinical relevance of a products' outcomes and the trade-offs patients are willing to make between the benefits and risks at the time of regulatory benefit-risk assessment and HTA (35). The initial phase of the PREFER project included discussions with a broad representation of stakeholders, for example, patients, patient organizations, regulatory authorities. HTA bodies and reimbursement agencies. These discussions highlighted interest from these stakeholders in preference studies but also the need to further explore and test methods and their usefulness for decision making (36). The recommendations from PREFER are expected to lead to changed practices, in that stakeholders, including industry, will routinely assess whether a preference study would add value at key decision points in the medicinal product life cycle and, if so, implement patient preference studies according to the PREFER project recommendations (37).

## 2. Materials and Equipment

This qualitative protocol was developed and described by adhering to the following guidelines for the use and reporting of qualitative research, attribute and level development: (i) the recommendations by Coast and colleagues on the use of qualitative data collection and analysis methods for attribute development (28), (ii) the steps concerning attribute and level development in health care preference research described by Bridges and colleagues (38), (iii) the criteria for good attributes described by Hensher (39), and (iv) the framework method for thematic analysis described by Lacey and Luff (40) (see “analysis and reporting” section). As recommended by Hollin, Coast, and Bridges (27), this protocol covers: (i) the rationale for the method used to develop attributes, (ii) the nature of the included sample in the focus group discussions, (iii) details on the nature of the sampling, (iv) the focus group guides, (v) who conducted the focus groups and in what setting, (vi) whether the focus groups were transcribed, and (vii) details of the analysis.

## 3. Methods

### 3.1. Step-by-Step Procedures

**Figure 1:**
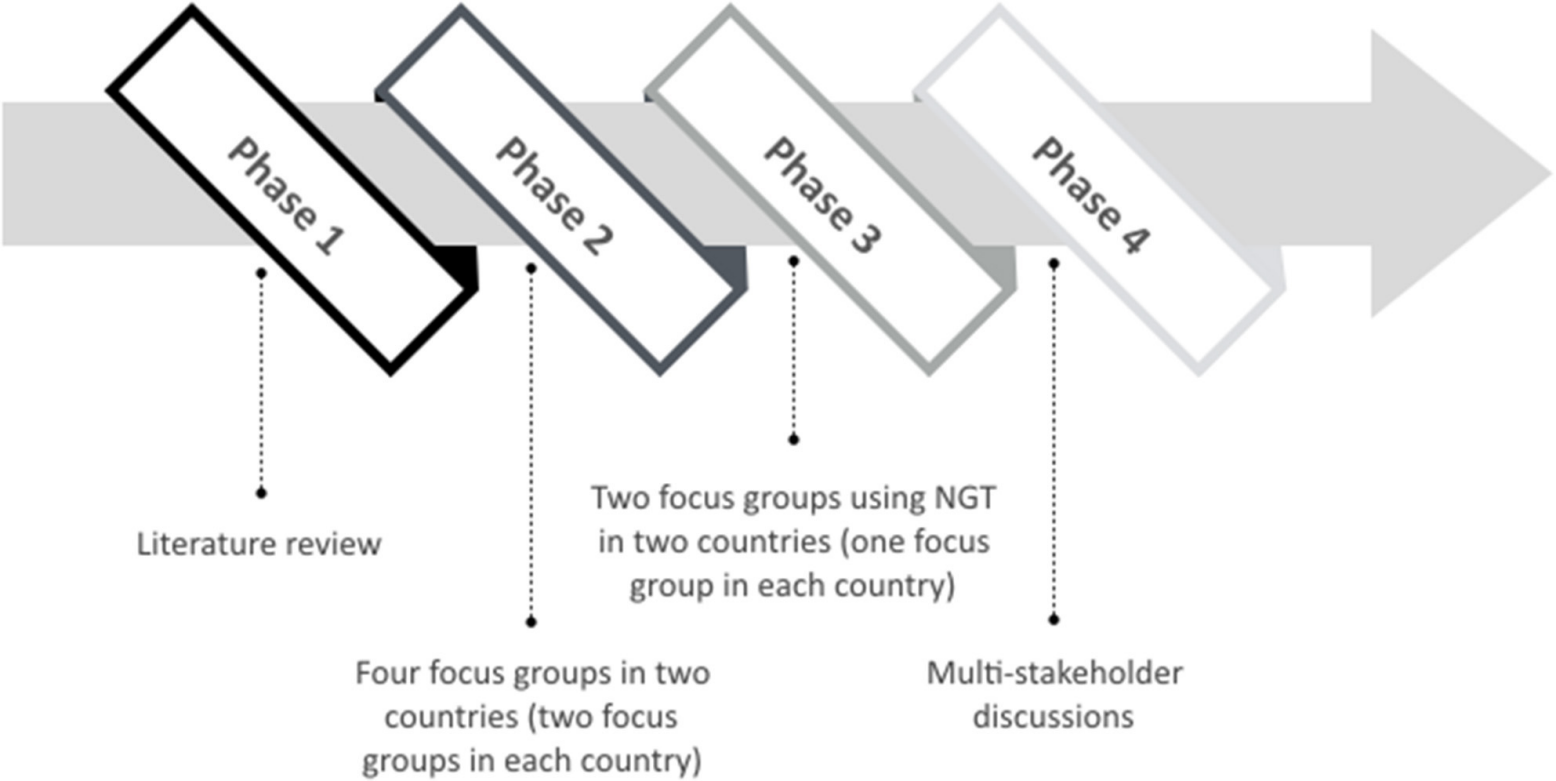
Four-staged qualitative study design.

Since there is limited recently published research regarding patient preferences for lung cancer treatment (including newer types of therapies such as immunotherapies), an extensive exploratory qualitative phase will be conducted involving different phases. Several authors recommend using qualitative methods with patients and performing a literature review to inform the attribute and level development (27, 28, 38, 41). Bridges et al. (38) describe that this process should be supported by evidence on the potential range and values that people may hold and that consultation with clinical experts, qualitative research or other preliminary studies can provide the basis for attributes and levels evaluated in preference surveys. Hilligsmann and colleagues conducted a *Nominal Group Technique* (NGT) in the context of drug choices and confirmed its usefulness to identify attributes for subsequent preference surveys. The authors describe that because of its advantages of being rigorous, systematic, and transparent, the use of NGT may improve the validity of subsequent preference surveys (41). Therefore, this study will involve the following four phases, with results from each phase informing the next phase (see Figure 1).

#### 3.1.1. Phase 1: Scoping Literature Review

In the initial phase of this study, a scoping literature review of patient preference studies in the lung cancer treatment setting will be performed to identify an initial list of treatment characteristics searching will be conducted in: (i) previously performed preference studies among lung cancer patients, (ii) benefits and risks of treatments already being prescribed to lung cancer patients, and (iii) treatment characteristics of medicines that are currently being studied in clinical trials for the treatment of lung cancer patients. Searches will be conducted in two electronic databases (Web of Science and PubMed), by using free text terms and *Medical Subject Headings* (MeSH). Since different electronic databases have different MeSH terms, the key search terms will be adapted to each specific database. Therefore, variations of the following key search terms will be adopted: “Lung Cancer” AND “Patient preferences.” Only research papers published in English will be considered. In case of reviews or meta-analyses, included original articles will be evaluated for inclusion in this scoping review. The results will be screened using a two-fold process. First, the title and abstract will be screened based on the inclusion criteria that the studies have to assess the treatment of lung cancer and assess patient preferences for these treatments. Afterwards, the full text of the selected article will be reviewed to ensure that the article will be relevant to the scoping review based on the above inclusion criteria. If the article meets the inclusion and exclusion criteria, it will be included in the review and information from the study will be extracted for analysis. The list of treatment characteristics emerged from this literature search will be used to trigger further discussion in the initial focus group discussions (see Phase 2).

#### 3.1.2. Phase 2: Initial Focus Group Discussions

The list of treatment characteristics emerged in the literature review (Phase 1) will be used in the second phase of this qualitative protocol: focus groups discussions to identify which treatment characteristics lung cancer patients find most relevant and why. Focus groups discussions were selected as the method for data collection instead of interviews because they allow for interactivity between participants, active discussions guided by the researchers, and thereby may generate topics that patients and researchers may not have recognized through other means. The choice for focus group discussions considered the recommendation by Coast and colleagues (28) that the choice between qualitative methods for attribute development may ultimately be determined by practical considerations such as the sensitivity of the topic.

Regarding representativeness, we envision that several patient characteristics, such as socio-demographics, type of cancer, staging and treatment experience may influence their opinions and we want to ensure that the particular attributes and levels identified in this study are not geared to only patients with a specific disease, treatment history or country of origin. Therefore, we aim to introduce heterogeneity in terms of country and include patients in different stages of their disease (III and IV), see “inclusion criteria” section. The scoping review of Phase 1 will help to increase the chance that the eventually found attributes are important to different types of patients, as it will identify the characteristics that will be evaluated by patients in the focus groups and will include characteristics from previously conducted preference studies in different countries in lung cancer patients, as well as side effects of products currently being administered to lung cancer patients across countries.

Participants will first be asked to complete an answer sheet to gather information on the socio-demographic background and health literacy, using Chew's Brief Literacy scale (42). We will aim to transparently describe and characterize the participants by means of the patient characteristics collected through this answer sheet. We will also include a transparent description of the methods used (including recruitment, setting of the focus group) when we describe the results. Further, in the survey following this study, we aim to include a larger population and we will describe the representativeness when we report the results through the characterization of patients using the same patient characteristics. Finally, in the quantitative survey based on this qualitative research, we will investigate the influence of several patient characteristics (socio-demographic data, treatment and disease experience) on their preferences.

Since our goal is to identify “core” attributes in lung cancer treatments, we estimate to conduct four focus groups in two different countries (two focus groups for each county). There are no clear guidelines on when “enough is enough” (43), although literature highlighted that some projects reach saturation after conducting 4–6 focus groups (43–45). Saturation is defined as the point when “no new information or themes are observed in the data” (46) (p. 59). In qualitative studies, data saturation occurs when redundancy is reached in data analysis and signals to researchers that data collection may cease (47). Hennink and colleagues (48) have underlined that few focus groups are enough to reach data saturation when the goal is to identify “core” issues. Thus, we expect that four focus groups will be enough to reach data saturation. If data saturation is not achieved with these focus groups, additional focus groups will be considered.

As part of the recruitment process, an invitation letter will be sent to those expressing interest in the study and fulfilling inclusion criteria (see “Participants” section). Those interested in participating will then be contacted by a member of the study team to verify their willingness to participate and if so, arrange the practicalities of the focus group discussion. A participant information sheet will be posted, emailed or given to participants prior to the discussion. At the start of the discussion, an informed consent procedure will take place and a consent form will be signed by the participants before proceeding with the focus group discussion. Each focus group is anticipated to last around 90 min to avoid excessive fatigue and will include a mid-session break of approximately 10 min.

Potential differences in moderating styles will be minimized by using a focus group guide (see Appendix A). Each focus group will be led by a team consisting of one moderator, one assistant, and/or one note-taker who have experience with qualitative research approaches and conducting focus group discussions. To increase the quality of the attribute development, the team members will also be involved in the subsequent quantitative preference survey.

Both bottom-up and top-down approaches will be used to develop attributes in these initial focus group discussions; patients will be first asked openly about which treatment characteristics matter most to them (= “bottom-up”) and only afterwards reflect on examples of treatment characteristics retrieved *via* the literature search described in Phase 1 (= “top-down”) in order to trigger further discussion.

All focus group discussions will be audio-recorded (with the participants' permission) and will later be professionally transcribed verbatim to a digital document with any identifiable data removed to preserve participant confidentiality.

#### 3.1.3. Phase 3: Additional Patient Focus Groups Using Nominal Group Technique

The aim of this phase is to refine and rank in a standardized manner the list of treatment attributes emerged in Phase 2 through the NGT (41, 49). The NGT method is specifically suitable for attribute development because it involves a ranking exercise and allows the identification of lung cancer treatment characteristics rated most highly by patients (28, 50). Compared to other qualitative consensus methods, NGT is more efficient in enabling groups to reach consensus quickly (28, 50). Additionally, the highly structured process minimizes the information loss that can sometimes occur with focus groups and responses are assumed to provide interpretable and valid ordinal data that reflect implicit prioritized views held by participants because equal weights are given to all group members (51, 52). The NGT method will be applied in two focus groups in two countries (one focus group in each country) and will consist of three steps:

(i) First, following the informed consent process, participants will be provided with a pre-developed list of characteristics generated by the previous focus groups (Phase 2). This will allow the participants to silently internalize the concepts to be discussed during the focus groups discussion. All treatment characteristics will also be orally explained by the moderator. Subsequently, each participant will be asked to individually rank the list of characteristics according to how important they found them (from most important to least important) and if they feel a particular characteristic is missing, they will have the opportunity to include this in their ranking sheet. The assistant will collect the individual ranking sheets once finished and determine a group score and rank order for each of the characteristics from the individual rankings.

(ii) In the second step, the group scores and rank order will be presented to participants, and a discussion will be held on the group scores and rank order. During the discussion, participants will be asked to reflect on how their individual rank order compares to the group rank order.

(iii) Finally, participants will have the opportunity to reconsider their initial ranking in light of the group discussion. They will be under no pressure to achieve consensus, and all rankings will again be made individually. As for Phase 2, all the focus group discussions will be audio-recorded (with participants' permission) and later transcribed to a digital document with any identifiable data removed for confidentiality. As for phase 2, potential differences in moderating styles will be minimized by using a guide (see Appendix B) and the moderator, the assistant, and/or the note-taker will have experience with qualitative research approaches and conducting focus group discussions.

#### 3.1.4. Phase 4: Multi-Stakeholder Discussions

In the final phase, discussions with oncologists, patient organization members, and stakeholders from different areas of medicine and scientific disciplines including preference research, psycho-oncology, oncology, health economics, drug development, pharmaceutical sciences, and biomedical sciences will be held. These discussions will aim to define each attribute based on the rank order and qualitative analysis of the focus groups, to identify and define the levels of each attribute and to reduce the number of the attributes, if necessary.

### 3.2. Participants

Guidance on focus groups' size is common and seldom goes beyond a minimum of 4 and a maximum of 12 participants per group (53–57). McMillan and colleagues (50, 58, 59) highlighted that groups of between 2 and 14 participants have generally been used in NGT research, and an average of seven participants for each group is recommended to collect a diversity of information and facilitate sufficient group interaction. On these bases, each focus group will consist of around seven NSCLC patients at stage III and IV. A much larger number would slow the staged process of the discussion that aims to reach consensus in a relatively short period of time (around 90 min).

All focus groups will be conducted in Italy and Belgium. These countries are chosen because they are characterized by differences such as unequal financing, service provision, and access to healthcare (60). This will allow researchers to understand which treatment characteristics are most important to lung cancer patients who live in countries who offer different kinds of healthcare systems (60–63). Specifically, Belgian insurances that cover healthcare expenses are compulsory and are chosen directly by citizens. Further, Belgium has a high level of health expenditure, a moderate level of inpatient healthcare, a high level of outpatient healthcare and patients have a high freedom of choice. Italian's healthcare system is mixed, public and private and is characterized by a medium level of total health expenditure. The system is financed by taxes directly paid by citizens to the state and by population and economic entities' contributions. Compared to Belgium, the level of inpatient healthcare providers is similar but the outpatient provider level is low. The access to doctors is highly regulated.

Italian participants will be recruited at the European Institute of Oncology in Milan and Belgian participants will be sampled at the University Hospital in Leuven. Patients will be recruited by the treating oncologists who will be able to evaluate their clinical and psychological status as well as their motivation to provide information on their preferences. Different patients from the one recruited in the second phase will be contacted and offered to participate in the third phase of the qualitative research.

The following eligibility criteria will be used:

#### Inclusion Criteria

- Adult patients (≥18 years old);
- In treatment patients with a histological or cytological diagnosis of NSCLC stage III or IV as classified by the *Union for International Cancer Control TNM Classification of Malignant Tumors* (UICC TNM VIII Edition). The reason for including NSCLC patients at stage III and IV is that late-stage patients often have received multiple types of treatments and are thus able to reflect on a broad range of different treatment characteristics, thereby increasing the chance that all relevant treatment characteristics will be identified.

#### Exclusion Criteria

- Cognitive impairment or inadequate verbal skills that may render them incapable of agreeing to participate in an informed and voluntary fashion (as evaluated by the clinician);
- Inability to understand study materials (as evaluated by the clinician);
- Physical or psychological impairment that prohibits their participation in the focus group (as evaluated by the clinician).

### 3.3. Analysis and Reporting

The audio-recordings will be transcribed verbatim in the language used in the focus group and then will be translated into English by a professional transcribing company. In the first set of focus groups (Phase 2), transcripts and notes from the focus groups will be thematically analyzed using an iterative approach as described in the framework method by Lacey and Luff (40) and summarized in Table 1. The thematic analysis is a “*method for identifying, analyzing, and reporting patterns (themes) within data”* (64) (p. 6) and will be used to generate a list of potential attributes. The analysis will follow the following recommendations for attribute and level development:

- An iterative, constant comparative analysis approach should be used to constantly modify and extend categories to ensure that all key aspects can be incorporated through this modification (28);
- Attributes should be relevant to patients and/or policymakers, relevant to the decision context, plausible and capable of being traded (28, 38);
- Attributes should include all those that might be important for an individual in coming to a decision, as ignoring important attributes may bias findings (28);
- Qualitative work to determine overarching attributes encompassing key themes combined with piloting should be used to avoid the above problem (28);
- Attributes should not be too close to the latent construct, for example, overall happiness with a product (28);
- Single attributes should not have such a large impact on decisions that large numbers of respondents essentially make no errors in decision-making (28);
- Attributes should not be intrinsic to person's personality; instead, such aspects that may determine preferences should be included as variables for investigating preference heterogeneity (28);
- Attribute development should be thought of as a process that consists of conceptual development where the attributes are identified, followed by refinement of language to convey the intended meaning to the participants of the preference survey (28);
- All attributes that potentially characterize the alternative treatments presented to participants in the preference survey (in this case different lung cancer treatments) should be considered, while considering that some may be excluded to ensure the alternative treatments are plausible to subjects (38);
- A good attribute meets the following criteria: realistic, plausible, relevant, tradable, clear and unambiguous, distinctly different from the other included attributes, comprehensive, and of salience to respondent' decisions (38, 39).

**Table 1:** Iterative steps of the framework method used in the thematic analysis of the initial four focus groups (Phase 2).

1. Familiarization	Researchers of each country involved in the study will thoroughly read and re-read the transcripts. They will use the margins of the transcripts to write down analytical notes, thoughts, or impressions (e.g., when focus group participants expressed exceptionally strong or contrasting views).
2. Identifying a thematic framework	To identify an initial thematic framework, four researchers will independently code the transcripts for each focus group, meaning that they will attach specific themes or concepts to particular paragraphs, based on the research aims of the study. These codes will be different factors, such as treatment outcomes, side effects, and symptoms patients mentioned during the focus groups.
3. Coding	Researchers will discuss these preliminary codes to assess whether they interpreted the focus group in the same manner and to reach a consensus about the final coding list. The final coding list (i.e., framework) will consist of the final list of attributes to be used for ranking in the final focus group. NVivo Software, version 11.0 will be used to code the transcripts using the final coding list.
4. Mapping and interpretation	Meetings will be organized between researchers involved in the study in order to discuss their interpretations. This process will take into consideration potential differences between the Italian and Belgian focus groups but also between the first two focus group discussions within each country.
5. Charting	The charting step will involve summarizing and reporting the data based on the themes identified through the analysis, as described by Lacey and Luff (40) and will be performed after the final two focus groups and multi-stakeholder discussions involving clinicians and preference experts.

Transcripts will be independently coded by researchers. These lists of attributes will then be compared and combined across sites to generate a comprehensive list of possible attributes for preference instrument development. In the second set of focus groups (Phase 3), the list of attributes will be prioritized using the NGT. During the NGT process, the individual rankings will be summed across participants to derive the rank order at the group level. To obtain a final rank order of characteristics, the mean for each of the treatment characteristics will be calculated by combining the two rank orders reached in the two countries.

## 4. Compliance with Ethical Standards

The study will be conducted according to the EU *General Data Protection Regulation* (GDPR). Additionally, this study was approved by the *Ethische Commissie Onderzoek UZ/KU Leuven* (Belgium; reference S63007) and the *Ethical Committee of the European Institute of Oncology IRCCS* (IEO, Milan, Italy; reference 1027/19-IEO 1093). An information sheet and informed consent form will be provided prior to conducting focus groups. The information sheet will inform participants that participation will not affect their healthcare, that participation is voluntary and that they can withdraw their consent at any time. Participants will have the opportunity to ask questions and to discuss concerns with researchers involved in the study. Written informed consent will be obtained without any coercion of study participants. Participants will be made aware that any identifiable information will be deleted and that their names will be replaced with codes (pseudonymized).

## 5. Dissemination

The findings of this study will be disseminated *via* international peer-reviewed journals and scientific conferences. A summary of the study results will also be written for the lay audience and made available to participants and relevant patient organizations for distribution on their own channels. Patient organizations will be approached to help to disseminate the publication to their members.

## 6. Anticipated Results

This study protocol will define a list of attributes and attribute levels that will inform the design of a quantitative preference survey. Additionally, this study will provide information relevant from the patient perspective:

1. A summary of insights obtained from focus group discussions with NSCLC patients at stage III and IV;
2. An identification of themes relevant to patients that will be evaluated in the quantitative phase of the study.

Understanding which treatment attributes patients find important may be especially relevant in lung cancer, where the existence of different (novel) lung cancer treatments with different benefits (e.g., regarding progression-free survival, overall survival, response rate), risks (e.g., fatigue, negative body perception) and other characteristics (e.g., route of administration and treatment schedule) creates uncertainty on the value of these treatment attributes according to lung cancer patients (65). Such uncertainty underlines the value of decision-making by drug developers, regulators, payers and clinicians that takes into consideration evidence from patient preference studies.

This research protocol will be useful to collect information on advanced lung cancer patient preferences. Results from such studies can also inform clinicians and healthcare providers of relevant factors on patient preferences and these characteristics can be incorporated in decision aids that aim to improve shared decision-making between patients and clinicians (12). Understanding what patients believe to be important attributes of their treatment and which risk(s) they are willing to tolerate, could facilitate medical decision-making and could also promote personalized decisions regarding the therapeutic approach and ensure a more precise and collaborative approach with patients (66–69).

This protocol can be used as a resource for drug developers as well as HTA and regulatory bodies who themselves can be interested in designing and conducting patient focus group discussions to enrich their decisions with patient values. The *European Medicines Agency* (EMA) has stated its intent to conduct disease-specific focus groups to include patient preferences in regulatory benefit-risk assessment (25). Another example concerns an exploratory preference study that received advice from the HTA body in the United Kingdom, the *National Institute for Health and Care Excellence (*NICE) (24, 70). This study aimed to determine how patient preference data could be used in HTA; the project consisted of a focus group with multiple myeloma patients to inform a subsequent preference survey. Learnings from this qualitative study can also inform the development of PREFER's evidence-based recommendations for future preference study developers and assessors on how to assess and use patient preference studies.

Additionally, in view of limited evidence from lung cancer patients regarding newer lung cancer therapies, we believe that the attributes identified through applying this study protocol may be informative for different healthcare stakeholders involved in the development, evaluation, and prescription of lung cancer treatments to understand the value of treatment outcomes as evaluated by advanced lung cancer patients. Specifically, these attributes may inform drug developers, researchers, and patient organizations on patient-centered drug development such as *via* the identification of patient-centered clinical trial endpoints and the development of so-called *Patient Reported Outcome Measures* (PROMs) in clinical trials. Finally, the use of qualitative and quantitative evidence on how important patients find different cancer treatment attributes in marketing authorization and reimbursement decision-making could add to the available clinical evidence on benefits and risks, already considered in these decisions, as well as complement existing decision criteria for marketing authorization and reimbursement.

## 7. Discussion

This protocol describes the four-steps approach of a qualitative study aiming to identify patient-relevant lung cancer treatment attributes and to understand which treatment characteristics are most important for advanced lung cancer patients through a qualitative methodology. The use of qualitative methods will allow transparently document and report the lung cancer patient preferences on treatments.

In this study, the attributes will be developed by adopting both bottom-up and top-down approaches: we will allow to transparently document and report lung cancer patient preferences for treatment characteristics matter most to them, before they are asked to reflect on examples of treatment characteristics retrieved *via* the literature review. Focus groups and the NGT will allow us to select those treatment characteristics found most important for patients and use these for developing the attributes in the subsequent quantitative preference survey.

## Data Availability Statement

The original contributions presented in the study are included in the article/Supplementary Material, further inquiries can be directed to the corresponding author.

## Ethics Statement

This study was reviewed and approved by the Ethische Commissie Onderzoek UZ/KU Leuven (reference S63007) and the Ethical Committee of the European Institute of Oncology IRCCS (IEO, Milan, reference 1027/19-IEO 1093). The participants will provide their written informed consent to participate.

## Author Contributions

ID and RJ drafted the manuscript. All authors provided substantial input during the study design, critically revised the manuscript, and read and approved the final manuscript.

## Conflict of Interest

MS is employed by Alexion Pharmaceuticals, Inc. EK is employed by Janssen Research and Development. The remaining authors declare that the research was conducted in the absence of any commercial or financial relationships that could be construed as a potential conflict of interest.
