# Supplementary material for: Patient Preferences for Lung Cancer Treatment: A Qualitative Study Protocol Among Advanced Lung Cancer Patients
Source: Front Public Health. 2021 Feb 5;9:622154. doi: 10.3389/fpubh.2021.622154 (PMC7900128; doi:10.3389/fpubh.2021.622154)
Supplement: Supplementary file 2 [file Table_2.DOCX]

**Appendix A**

**Initial Focus Group Discussion Guide (Phase 2)**

**I. Preparations**

1. During recruitment

- Book a venue/meeting room that is neutral and easily accessible to patients and that allows for a circle seating around a table
- Provide the participants with instructions to get to the venue and room
- Provide your/assistants’ phone number(s) to the participants
- Print the information sheet, informed consents and forms with demographic questions^^[[1]](#footnote-0)^^

2. Day of the focus group

- Have enough information sheets, consent forms and forms with demographic questions with you
- Seating arrangements: circle seating around table
- Provide writing material and paper
- Provide name cards (mentioning only the first name of participants), to put on the table
- Provide water/coffee/tea + something to eat
- If slides^^[[2]](#footnote-1)^^ are available, set up slide show
- Take two audio recorders (+ extra batteries) with you and place these on the table at opposite sides
- Dress code for moderator and assistant(s): casual, pants and blouse is OK, no suits, no suit jackets/blazers, not too formal

3. Moderator and assistant team

● The moderator and/or assistant should be trained qualitative researchers, experienced with conducting focus group discussions

● The moderator and assistant team will consist out of different persons in the Belgium vs Italy focus group to enable the focus group discussion to take place in the native language of participants

● To ensure comparability between the focus group between the two teams and in order to prepare for the focus group, a meeting between moderator and assistant of both teams should be planned before the focus group to go over the guide and discuss the moderator and assistant’s tasks. This meeting will also discuss how the assistant should take notes and use these notes at the end of the focus group to summarize what has been said (see 3.2)

3.1 Attitude of moderator

● Exercise mild unobtrusive control (moderate the discussion but do not interrupt too often)

● Adequate knowledge of topic

● Appears like the participants

● Use purposeful small talk

● Alert and free from distractions (put sound of phone off and do not have your phone on you)

● Have the discipline of listening and apply active listening:

o Verbal reactions:

▪ Short verbal responses (e.g. “I see”, “Yes”, “Okey”, avoid "that's good", "excellent")

▪ Use pauses and probes

● 5 second pause probes: "Would you explain further?" "Would you give an example?"

▪ Listen for inconsistent/vague/cryptic comments and probe for understanding, e.g. "I don't understand."

▪ Consider asking a final yes/no question

▪ Uses probes to refocus the discussion when the discussion goes off-topic, e.g. “Now that we have talked about XXX, I would go back/address the question/topic…”)

o Nonverbal reactions:

▪ Head nodding

● Familiar with questioning route (know this protocol very well)

● Take into account the different types of participants and try to balance the conversation while addressing the obligatory topics: dominant talkers, shy participants, etc.

3.2 Tasks of the assistant:

- Handles logistics (location of refreshments, bathrooms, emergency exits)
- Collects consent forms and surveys
- Takes careful notes on paper or on laptop:
  - Anticipate that others will use your notes. Notes sometimes are interpreted days or weeks following the focus group when memory has faded. Consistency and clarity are essential.
  - It is essential that this information is easily identified and organized. The notes should be divided according to the focus group questions/topics and include time indications
  - Your notes will contain different types of information:
    - Quotes:
      - Listen for notable quotes, the well said statements that illustrate an important point of view. Listen for sentences or phrases that are particularly enlightening or eloquently express a particular point of view. Place name or initials of speaker after the quotations. Usually, it is impossible to capture the entire quote. Capture as much as you can with attention to the key phrases. Use three periods ... to indicate that part of the quote was missing.
    - Key points and themes for each question
      - Typically, participants will talk about several key points in response to each question. These points are often identified by several different participants. Sometimes they are said only once but in a manner that deserves attention. At the end of the focus group the assistant moderator will share these themes with participants for confirmation.
    - Follow-up questions that could be asked
      - Sometimes the moderator may not follow-up on an important point or seek an example of a vague but critical point. The assistant moderator may wish to follow-up with these questions at the end of the focus group.
    - Big ideas, hunches, or thoughts of the recorder
      - Occasionally the assistant moderator will discover a new concept. A light will go on and something will make sense when before it did not. These insights are helpful in later analysis.
    - Other factors
      - Make note of factors which might aid analysis such as passionate comments, body language, or non-verbal activity. Watch for head nods, physical excitement, eye contact between certain participants, or other clues that would indicate level of agreement, support, or interest. Nonverbal: movements, attitudes, emotions. These will be indicated in the notes by using a different color our by highlighting the text describing nonverbal reactions
  - These notes will be used by the assistant at the end of the focus group discussion to summarize the focus group (see end guideline)
- Controls for equal participation by all participants and informs the moderator if some participants are not getting the chance to participate
- Monitors audio recording equipment
- Time management via discrete signs to the moderator
- Gives a general summary based upon the above mentioned notes at the end of the focus group. The summary should encompass the different questions/topics addressed in the discussion and give a balanced view of the different opinions expressed

**II. Focus group agenda**

Everything below in *italic* is to be said to the participants, everything in black is guidance and can be told to the participants in your own words. In **bold** an indication of timing of actions is given; however, this is an indication of time, it is more important to finish the topics than to rush through the focus group.

00:00 Welcome the participants while they arrive

● Create warm and friendly environment

o Provide coffee and tea

o Interact with participants, and stimulate interaction between participants

● Make circular seating arrangements for participants according to their needs

● Provide the information sheet and let them fill in the consent form and survey

00:10 Check whether all participants have arrived. If not, the assistant will try to reach these persons via telephone, in a separate room. If the missing participants cannot be reached, the focus group will start without these persons. Let the arrived participants fill in the necessary forms and start the focus group with a general introduction:

● *Welcome, my name is* (your first name) *and I will be your moderator today. In addition, I brought* (first name of assistant) *to help me with the focus group*

● *My role as moderator will be to guide the discussion*

● *The discussion that we will have today is about what you think about medical treatments for lung cancer and what you expect from lung cancer treatments. We want to learn from you what you would value in a lung cancer treatment, what would make that you take or not take a lung cancer treatment and why.*

● *We want to have this discussion with you as patient, since you are the potential user of the treatment*

● *The opinions collected today will be used to develop a survey that we will spread out to a large amount of lung cancer patients. This will allow us to quantify (put in numbers) the opinions we collect today*

● *The opinions collected today will be not be used to change anything about your ongoing or future treatment*

● *In the end, we hope that the opinions we collect in this research will be used by pharmaceutical companies to develop lung cancer treatments tailored to patient needs, and by health authorities to make decisions on whether a specific lung cancer treatment can become available on the market or whether it can be reimbursed*

● *This study is part of a large European project called PREFER. This research project looks at how and when patient preferences for new treatments should be incorporated into the drug development process. PREFER aims to make the development of drugs and decisions about drugs more patient-centered.*

● *The focus group will take about 1.5 hour*

● *There will be a break in the middle of the discussion of 5-10 minutes*

00:12 Explain the “rules”:

● *There are no right or wrong answers, only differing points of view*

● *We are looking for your opinions and hope for a nice discussion*

● *It is possible that you do not agree with all opinions, but it would be nice if you could listen respectfully to each other*

● *Since this is an informal discussion, we will address each other only by their first name as indicated on the name cards*

● *We ask you to turn off the sound of your phones*

● *If there are any questions or terms that are used during the focus groups that are not clear to you, please let us know*

● *To be able to fully focus on the focus group as moderator and assistant, we will audio record our conversation today. Only researchers on the project will have access to the recordings. These recordings will be eventually destroyed after being transcribed into a computer file when identifiable data like names and date of birth will be deleted and your responses will be recognized just by a code. More details regarding the data coding and processing appear on your information sheet. If you do not want that your opinions are being audio recorded, you should not sign the information sheet nor choose to participate in the focus group*

● *To be able to fully understand what everybody says and also to help our analysis, it would be very helpful for the analysis if only one person is speaking at a time*

● *Are there any questions about what I just mentioned to you?*

● *We will now start the recording. Is that OK for everybody?*

00:15 *To get to know each other, we would like to do a round-the-table where each of us tells us shortly* (max 1 min each) *a little bit more about themselves (name, background) and why you decided to join today’s discussion.*

00:30 *As mentioned in the beginning, the discussion that we will have today is about what you think about medical treatments for lung cancer and what you expect from lung cancer treatments. We want to learn from you what you would value in a lung cancer treatment, what would make that you take or not take a lung cancer treatment and why.*

*Therefore, we would like to know: when you undergo a treatment for lung cancer, what type of improvement do you expect from it? With improvement we mean benefits or desirable effects.*

● *Why?*

*Some examples of improvements could be* ***(placeholder for examples retrieved via the literature)***

● *Are there any other types of improvements you can think of?*

● *Why are these important to you?*

00:45 Break. *We will now have a break of 10 minutes. Please feel free to use the restroom, take water/coffee/tea/something to eat.*

00:55 *Until now we discussed what benefits you expect from lung cancer treatments. Lung cancer treatments may also be associated with side-effects. With side effect we mean risks or undesirable effects of the treatment. When you undergo a treatment for lung cancer, what type of side-effects would make you want to doubt whether you want to keep on taking the treatment?*

● *Why?*

*What type of these side-effects would make you stop taking the treatment?*

● *Why?*

*Some examples of side-effects could be* ***(placeholder for examples retrieved via the literature)***

● *Are there any other types of side-effects you can think of?*

● *Why are these important to you?*

01:10 *We have now discussed types of improvements and side-effects that would influence your choice to take a lung cancer treatment. Thinking about the improvements and side-effects we just discussed:*

● *What type of side-effects would make you want to reconsider whether you want to continue the treatment?*

● *What type of improvements would make you want to accept more of the side-effects we just talked about?*

01:25 *Are there any other aspects of lung cancer treatment, besides the different side-effects and improvements we just talked about that would influence your choice to take or stop taking a lung cancer treatment? (open question)*

● *Why?*

*Some examples of other treatment characteristics could be* ***(placeholder for examples retrieved via the literature)***

● *Are there any other treatment characteristics you can think of?*

● *Why are these important to you?*

01:40 Finish. *The focus group is now finished*

● Summarize the lessons learnt of today’s discussion – by assistant

● Ask if the summary is correct, or if you have forgotten something

● Ask if there are any questions

● Thank all participants for their participation

Collect the following materials:

● Consent forms

● Short surveys

● Recordings

● Notes

1. *Including questions about general characteristics, clinical characteristics and health literacy questions (*Chew’s Brief Literacy scale [31]) [↑](#footnote-ref-0)
2. *Slides would include the questions in large font type and more information (e.g. explanation of treatment characteristics)* [↑](#footnote-ref-1)
